# The effect of childhood stunting and wasting on adolescent cardiovascular diseases risk and educational achievement in rural Uganda: a retrospective cohort study

**DOI:** 10.1080/16549716.2019.1626184

**Published:** 2019-06-24

**Authors:** Gershim Asiki, Robert Newton, Lena Marions, Anatoli Kamali, Lars Smedman

**Affiliations:** a Department of women’s and children’s Health, Karolinska Institutet, Stockholm, Sweden; b Medical Research Council/Uganda Virus Research Council, Uganda Research Unit on AIDS, Entebbe, Uganda; c African Population and Health Research Center, Health and systems for Health Unit, Nairobi, Kenya; d Department of Health Sciences, University of York, York, UK; e Department of Clinical Science and Education, Karolinska Institutet, Stockholm, Sweden; f Africa Program, International AIDS Vaccine Initiative, Nairobi, Kenya

**Keywords:** Child hood undernutrition, blood pressure, schooling, adolescence, Uganda

## Abstract

**Background**: Little is known about the long-term effects of early childhood undernutrition on adolescent cardiovascular disease risk and educational performance in low-income countries. We examined this in a rural Ugandan population.

**Objective**: To investigate if stunting or wasting among children aged 2–5 years is associated with cardiovascular disease risk or educational achievement during adolescence.

**Methods**: We conducted analyses using data from a cohort of children followed from early childhood to adolescence. Weight and height were measured in 1999–2000 when the children were 2–5 years of age and repeated in 2004/2005 and 2011. We compared cardiovascular disease risk parameters (mean blood pressure, lipids, HbA1c) and schooling years achieved in 2011 among 1054 adolescents categorised into four groups: those who experienced stunting or wasting throughout follow-up; those who recovered from stunting or wasting; those who were normal but later became stunted or wasted; and those who never experienced stunting or wasting from childhood up to adolescence. We controlled for possible confounding using multiple generalised linear regression models along with Generalised Estimating Equations to account for clustering of children within households.

**Results**: Wasting was negatively associated with systolic blood pressure (−7.90 95%CI [−14.52,-1.28], p = 0.02) and diastolic blood pressure (−3.92, 95%CI [−7.42, −0.38], p = 0.03). Stunting had borderline negative association with systolic blood pressure (−2.90, 95%CI [−6.41, 0.61] p = 0.10). Recovery from wasting was positively associated with diastolic blood pressure (1.93, 95%CI [0.11, 3.74] p = 0.04). Stunting or wasting was associated with fewer schooling years.

**Conclusion**: Recovery from wasting rather than just an episode in early childhood is associated with a rise in blood pressure while educational achievement is compromised regardless of whether recovery from undernutrition happens. These findings are relevant to children exposed to undernutrition in low-income settings.

## Background

In 2017, an estimated 155 million children under five years of age were stunted and 52 million were wasted globally: 38% of the stunted and 27% of the wasted children were from Africa [1]. Available literature shows that even in its mildest forms undernutrition in childhood could be associated with an increased risk of cardiovascular disease in adulthood [2–10]. Barker and colleagues first observed that undernutrition during fetal life, infancy and early childhood could permanently change the structure and function of the body through a phenomenon known as ‘programming’ [11]. The first study undertaken among 10,636 men born between 1911 and 1930 who were followed to adulthood showed that mortality from coronary heart disease was higher among those who had low birth weight [12,13]. Barker and Hales through the so-called ‘thrifty phenotype hypothesis’ also used developmental programming to explain the association between low birth weight and type 2 diabetes [14,15]. Undernutrition has also been reported to have long-term effects on cognitive development through several mechanisms including irreversible structural damage to the brain by reduction of myelin, an increase in neuronal mitochondria, a reduction in cortical dendrites in neural spines and a reduced ratio of granule to Purkinje cell in the cerebellum thus impairing infant motor development [16–18]. Fernald and colleagues demonstrated that stunting is associated with altered hypothalamic-pituitary-adrenocortical activity with raised cortisol levels, heart rates and urinary epinephrine which may lead to reduced cognitive ability among school-age children [19]. A study in Ethiopia showed that stunting and underweight among school children were associated with low levels of academic performance [20]. Few studies relating early childhood undernutrition with later life effects have been conducted in low- and middle-income countries (LMICs). Most studies relating undernutrition in childhood with adult health outcomes have been conducted in high-income countries, and these studies in high-income countries involved a period of undernutrition followed by recovery, a phenomenon referred to as ‘catch-up growth’ [21–23]. In LMICs where there is a double burden of undernutrition and obesity, some children who experience undernutrition may not recover as seen in high-income countries. A systematic review of studies from LMICs (only one from Africa) showed that undernutrition in childhood is a risk factor for high glucose concentrations, increased blood pressure, and harmful lipid profiles in adulthood [8]. However, taken together, the results from LMICs show weaker associations between birthweight and weight at infancy and chronic diseases. These could be explained by less catch-up growth experienced in LMICs or lack of nutrition transition in the LMICs.

Only rarely have studies tracing individuals from childhood to adolescence examined the influence of childhood undernutrition on cardiovascular disease and educational performance. The adolescent period is critical for identification of cardiovascular disease risk and for timely prevention of complications in adulthood [8,21]. Studies on the adolescent consequences of undernutrition in low-income countries are of particular interest because about half of all adolescents experience some degree of stunting and wasting during childhood and may differ in terms of recovery from undernutrition.

We hypothesized that adolescents who experienced undernutrition in childhood and recovered might have different cardiovascular diseases risk or educational outcomes compared to those who never recovered from childhood undernutrition (stunting or wasting). One of the novel things we did in our study was to disaggregate the children into four categories: First, children who were stunted or wasted while under five years and remained so up to adolescence; secondly, children who were stunted or wasted under five years but recovered by adolescence; thirdly those who were not stunted/not wasted and remained so up to adolescence and lastly those who were not stunted or wasted under five years of age but deteriorated to stunting or wasting during adolescence. This approach did not simply look at whether the child was stunted/wasted at one point in childhood but the change in their nutritional status by adolescent period as well. We postulated that these groups of children could end up with different outcomes at adolescence.

The Kyamulibwa General Population cohort (GPC) in rural south-western Uganda provided rich longitudinal data to test this hypothesis among adolescents whose anthropometric measurements were taken during childhood and repeated during adolescence along with cardiovascular disease risk and educational outcomes assessment.

## Methods

### Study design and setting

This was a retrospective cohort study involving linkage of data for adolescents who had their anthropometric measurements taken between 1999 and 2001 when they were aged two to five years and cardiovascular diseases risk and educational achievement data collected in 2011 at the age of 13–16 years. These children were enrolled in a general population cohort (GPC) in rural Uganda. The GPC was established by the British Medical Research Council (MRC) in 1989 mainly to study HIV infection trends and risk factors in rural Uganda [24]. The GPC had approximately 22,000 residents of 25 neighbouring study villages plus one village reserved for pilot studies located in a rural district which is about 120 km west of the capital city (Kampala). The main economic activity is small-scale subsistence farming. Literacy levels are low and people live in semi-permanent buildings made of locally available materials such as mud and wattle walls and grass thatched or tinned roofs. The predominant ethnic group, Baganda constitutes 75% of the population, 15% are migrants from Rwanda and 10% are ethnic groups from other parts of Uganda. In brief, annual rounds of surveys preceded by mapping and census have been conducted in the study area since 1989. All households within the study area were visited and consenting residents aged 13 years and above were interviewed in their homes by trained field workers. Every three years, all children below 13 years were included in a survey in which anthropometric measurements were taken.

### Data collection

#### Child surveys (2001-2005)

Three child surveys with anthropometric assessments were conducted in 1999–2000, 2001–2002 and 2004–2005, during which informed consent was sought from the child’s parent or carer and assent from the children aged 8–13 years before an interview with the parent or carer. During the interview, the child’s age was estimated from recalled date of birth by the mother or using immunization cards and baptism records. The weight for children aged 0–2 months was recorded using digital scales (Seca productions). A Salter spring was used for those aged 2–12 months with a pouch suspended from a firm bar and for children more than one year old who could stand, a spring balance weighing scale was used. All weight measurements were made to the nearest 0.1 kg. For height measurement, length for young children unable to stand was measured using 100 cm rod while lying horizontally. For children able to stand, a standing rod was used with one end of the rod placed on a flat surface, on which the child stood with bare feet, with the heels, back and buttocks touching the rod, and arms hanging at the sides. A flat board was placed at 90 degrees to the rod lightly compressing the child’s hair and his/her height was read to the nearest 0.1 cm.

#### Adult survey (2011)

In 2011, all residents aged 13 years and above were examined for some selected cardiovascular disease risk factors. The WHO STEPwise Approach to Surveillance questionnaire [25] was adapted for the study. Socio-demographic data including levels of education, ethnicity, and religion and dwelling conditions (wall type) were also collected. Data on consumption of starch, fruit and vegetables, alcohol, tobacco smoking, and physical activity were gathered through a self-report. Height was measured using the Leicester stadiometer to the nearest 0.1 cm and weight measured using the Seca 761 mechanical scales to the nearest 1 kg with participants wearing only light clothing and no shoes. Blood pressure was measured on the right arm using an appropriate cuff with a digital Omron M4 in the sitting position, taken three times, 5 min apart and the mean of the second and third reading was recorded. Biochemical analyses were performed using the Cobas Integra 400 plus chemistry analyser to determine HbA1c from whole blood samples and high-density lipoprotein cholesterol (HDL-C), Total cholesterol (TC) and triglycerides (TG) from serum samples. Low-density lipoprotein (LDL-C) was estimated by modified Friedewald formula [26]: LDL-C = TC-(HDL-C + TGX0.16) mmol/L. Educational performance was measured by the number of schooling years achieved during adolescence in 2011 as used in the publication of Friedman and colleagues [27].

### Statistical analysis

We used SAS 9.4 (SAS Institute Inc., Cary, NC, USA) for the analyses. The childhood nutritional status data were derived by combining the 1999–2000 and the 2001–2002 data as baseline measurements. We then merged this file with the files from the 2004–2005 and 2011 surveys using the unique identity numbers assigned to each individual in the census or at birth registration. We included only children who were available in all these data collection time points; those first examined below five years of age and had their weight and height recorded in the follow up of 2004–2005 and 2011. Those missing all outcome variables (blood pressure, lipids, HbA1c, or years of schooling) in 2011 were excluded from analysis, but if they had at least one outcome variable, they were included. In order to obtain nutritional status, we derived z-scores for height-for-age and weight-for-height using the WHO-igrow SAS macro based on WHO growth standards 2006 for children under five years [28] and WHO growth reference 2007 for those aged 5–19 years. Stunting was defined as height-for-age < −2.0 z score, and wasting was defined as weight-for-height < −2.0 z score. Individuals with extreme values of z scores for height-for-age (z-score <−6.0 or >6.0) and weight-for-height (z-score <−5.0 or >5.0) considered to be biologically implausible were excluded from analysis. We categorised children into four groups, respectively, for height-for-age and weight-for-height z scores measured during childhood/baseline, and adolescence/follow-up. The first group referred to as ‘normal’ were children whose z scores were normal (−2.0 to 2.0) in the entire follow-up period. The second group named ‘stunted’ or ‘wasted’ were those with z scores below normal (< −2.0 z scores) throughout the follow-up period. The third group labelled as ‘recovered’ were those with z scores below normal at baseline but recovered to normal during follow-up and the fourth group labelled as ‘deteriorated’ were normal at baseline but fell below normal during follow-up.

All the outcomes (blood pressure, blood lipid levels, HbA1c and years of schooling) were treated as continuous variables and examined for deviations from symmetrical Gaussian distribution using tests of skewness, and by observing their histograms. Since there was no evidence of skewness, a generalised linear model was used to compare mean values of the outcomes for the four independent groups (normal, stunted, recovered, deteriorated) respectively for height-for-age and weight-for-height. Multiple generalised linear regression analyses were used to examine the relationship between each of the outcomes and the nutritional categories of children defined above and other covariates if shown to have association at p < 0.10 in the bivariate analysis. Age, sex and socio-economic factors were added as *a priori* factors into all the models. Since some households had more than one child enrolled, clustering of children within households was adjusted for using Generalised Estimating Equations (GEE).

## Results

### Study population characteristics

In total, 1,054 (79%) of the children who were available in the 1999–2002 baseline survey were found in the 2011 survey as adolescents. In the baseline assessment when the children were below 5 years of age, 39.2% were stunted, 15.5% had wasting and 5.4% had both. Table 1 shows how the nutritional status of the children changed as they progressed from childhood to adolescence and their characteristics during adolescence. Overall, 261 (28.3%) maintained a normal height-for-age while 93 (10.1%) remained stunted throughout the follow-up. At follow-up, 295 children (31.5%) had recovered from stunting and 275 (29.8%) who were initially normal had become stunted (deteriorated). Regarding the weight-for-height parameter, 12 children (1.3%) remained wasted, 245 (15.6%) had recovered from wasting and 307 (33.1%) had become wasted (deteriorated) later during follow-up and 464 (50%) remained normal.

**Table 1.** Characteristics of Adolescents in rural Uganda stratified by nutritional status during childhood.

		Change in height-for-age									Change in weight-for-height							
		None				Changed					None				Changed			
		*Normal*		*Stunted*		*Recovered^1^*		*Deteriorated^1^*			*Normal*		*Wasted*		*Recovered^2^*		*Deteriorated^2^*	
Characteristic		*n*	%	*n*	%	*n*	%	*n*	%		*n*	%	*n*	%	*n*	%	*n*	%
Total		261	28.3	93	10.1	295	31.5	275	29.8		464	50.0	12	1.3	145	15.6	307	33.1
Age at adolescence																		
	13–15	137	52.5	60	64.5	147	49.8	165	60.0		247	53.2	9	75.0	70	48.3	183	59.6
	16–17	61	23.4	15	16.1	88	29.8	70	25.5		118	25.4	3	25.0	39	26.9	72	23.5
	18–19	63	24.1	18	19.4	60	20.3	40	14.6		99	21.3	0	0.0	36	24.8	52	16.9
Sex																		
	Male	108	41.4	63	67.7	128	43.4	151	54.9		215	46.3	7	58.3	65	44.8	166	54.1
	Female	153	58.6	30	32.3	167	56.6	124	45.1		249	53.7	5	41.7	80	55.2	141	45.9
Tribe																		
	Muganda	179	68.6	74	79.6	224	75.9	203	73.8		348	75.0	9	75.0	98	67.6	234	76.2
	Other	82	31.4	19	20.4	71	24.1	72	26.2		116	25.0	3	25.0	47	32.4	73	23.8
Education level attained																		
	Incomplete primary	115	47.5	70	75.3	133	46.7	153	57.5		219	49.9	9	75.0	69	49.6	174	58.4
	Completed primary	53	21.9	8	8.6	56	19.7	49	18.4		84	19.1	1	8.3	29	20.9	47	15.8
	Post-primary	74	30.6	15	16.1	96	33.7	64	24.1		136	31.0	2	16.7	41	29.5	77	25.8
Religion																		
	Roman catholic	150	60.0	60	65.9	168	59.4	170	64.4		281	62.9	9	75.0	86	61.0	176	59.7
	Muslim	68	27.2	24	26.4	75	26.5	66	25.0		118	26.4	2	16.7	31	22.0	84	28.5
	Other Christian	32	12.8	7	7.7	40	14.1	28	10.6		48	10.7	1	8.3	24	17.0	35	11.9
Wall construction material																		
	Mud and pole	32	17.3	17	25.0	44	21.9	36	19.3		66	20.1	1	12.5	20	19.4	44	21.1
	Mud and brick	90	48.7	23	33.8	82	40.8	84	44.9		153	46.7	5	62.5	42	40.8	87	41.6
	Concrete and brick	63	34.1	28	41.2	75	37.3	67	35.8		109	33.2	2	25.0	41	39.8	78	37.3
Roof quality																		
	Poor	28	15.1	12	17.7	32	15.8	24	12.8		56	17.0	4	50.0	15	14.4	27	12.9
	Fair	80	43.0	22	32.4	63	31.2	66	35.3		115	35.0	4	50.0	37	35.6	86	41.2
	Good	78	41.9	34	50.0	107	53.0	97	51.9		158	48.0			52	50.0	96	45.9
Fruit/Vegetable consumed																		
	4 or less servings/day	169	64.8	54	58.1	173	58.6	170	61.8		288	62.1	7	58.3	85	58.6	197	64.2
	5 or more servings/day	92	35.3	39	41.9	122	41.4	105	38.2		176	37.9	5	41.7	60	41.4	110	35.8
Starchy staples consumed																		
	1–4 servings/day	76	29.1	20	21.5	52	17.6	63	22.9		115	24.8	2	16.7	30	20.7	67	21.8
	5–9 servings/ day	123	47.1	43	46.2	150	50.9	142	51.6		228	49.1	6	50.0	79	54.5	163	53.1
	10+ servings/day	62	23.8	30	32.3	93	31.5	70	25.5		121	26.1	4	33.3	36	24.8	77	25.1
Ever taken alcoholic drink																		
	No	239	91.6	86	92.5	270	91.5	249	90.6		427	92.0	12	100.0	130	89.7	280	91.2
	Yes	22	8.4	7	7.5	25	8.5	26	9.5		37	8.0			15	10.3	27	8.8
Physical activity																		
	None	65	24.9	18	19.4	88	29.8	68	24.7		118	25.4	1	8.3	37	25.5	79	25.7
	1–4 days/week	102	39.1	34	36.6	113	38.3	96	34.9		183	39.4	4	33.3	53	36.6	109	35.5
	5 or more days/week	94	36.0	41	44.1	94	31.9	111	40.4		163	35.1	7	58.3	55	37.9	119	38.8
HIV status																		
	Negative	236	90.8	83	89.3	270	92.2	244	88.7		417	90.3	10	83.3	130	89.7	278	90.9
	Positive	24	9.2	10	10.8	23	7.9	31	11.3		45	9.7	2	16.7	15	10.3	28	9.2

Recovered^1^ means child was stunted during pre-school years and normal from 7 years.

Rocovered^2^- child was wasted in pre-school years and normal from 7 years.

Deteriorated^1^ means child was normal during pre-school years and became stunted from 7 years.

Deteriorated^2^- child was normal in pre-school years and became wasted from 7 years.

The majority of children in the sample were young adolescents aged 13–15 years, of the main ethnic group (Baganda), with incomplete primary education, and of Roman Catholic faith. Approximately one-third of the adolescents lived in houses made of brick and concrete walls considered to be the best dwelling condition in the study population. The majority of adolescents consumed large quantities of starchy staples per day and less than half consumed adequate (five servings per day) of fruit or vegetables. Alcohol consumption was reported by about 7–10% of the adolescents. HIV prevalence was 10%. Boys dominated the category of children who remained stunted or wasted throughout follow-up or deteriorated later in their nutritional status. Children who were stunted or wasted all through or those who became stunted later had higher HIV prevalence (Table 1).

### Distribution of adolescent cardiovascular disease risk factors and schooling achieved, by nutritional status change from childhood to adolescence

Table 2 shows mean values for blood pressure, lipid levels, HbA1c and years of schooling for categories of children with their nutritional status change over time. Children who were stunted or wasted throughout the follow-up period had the least mean values for blood pressure and years of schooling. Those who were wasted throughout follow-up had the least mean values for TGs and HbA1c and higher mean of TC and HDL-C. Those who recovered from wasting had higher mean for diastolic pressure. Years of schooling were least among those who were stunted or wasted throughout, followed by those who recovered and then those who deteriorated later and normal children. Those who experienced both forms of undernutrition but recovered later had higher mean blood pressure and fewer school years than normal children.

**Table 2.** Distribution of adolescent cardiovascular disease risk factors and schooling outcomes by nutritional status.

		Mean blood pressure (mmHg)			Mean Lipid level (mmol/L)				
		Systolic	Diastolic	TC	TGs	HDL-C	LDL-C	HBA1c (%)	Schooling years
Nutritional status	Number	Mean (sd)	Mean (sd)	Mean (sd)	Mean (sd)	Mean (sd)	Mean (sd)	Mean (sd)	Mean (sd)
**Height-for-age**									
Normal	261	119 (12.0)	70.4 (8.3)	3.6 (1.0)	1.2 (0.7)	1.0 (0.4)	2.0 (0.8)	3.8 (1.3)	9.0 (8.8)
Stunted	93	115 (11.5)	68.9 (8.8)	3.7 (0.9)	1.2 (0.5)	1.0 (0.3)	2.1 (0.7)	3.7 (0.6)	5.8 (3.0)
Recovered	295	118 (11.2)	70.1 (8.5)	3.7 (1.0)	1.2 (0.6)	1.0 (0.4)	2.1 (0.8)	3.7 (0.7)	8.4 (6.4)
Deteriorated	275	118 (11.9)	69.8 (8.5)	3.5 (0.9)	1.1 (0.5)	1.0 (0.4)	2.0 (0.7)	3.7 (0.6)	8.5 (11.5)
**Weight-for-height**									
Normal	464	118 (12.3)	70 (8.1)	3.6 (1.0)	1.2 (0.6)	1.0 (0.4)	2.1 (0.8)	3.7 (1.1)	9.2 (11.0)
Wasted	12	111 (9.2)	67 (4.2)	3.8 (1.1)	1.1 (0.5)	1.2 (0.4)	2.1 (0.9)	3.6 (0.5)	6.2 (2.5)
Recovered	145	119 (9.7)	72 (8.4)	3.7 (1.0)	1.2 (0.6)	1.0 (0.4)	2.1 (0.8)	3.7 (0.7)	7.8 (3.8)
Deteriorated	307	117 (11.2)	69 (8.2)	3.5 (0.9)	1.2 (0.5)	1.0 (0.4)	2.0 (0.7)	3.7 (0.6)	7.6 (6.3)
**Experienced both^a^**									
Normal	269	117.7 (12.4)	69.8 (7.9)	3.6 (0.9)	1.2 (0.7)	1.0 (0.4)	2.0 (0.7)	3.7 (1.2)	9.8 (13.0)
Recovered	20	118.8 (9.7)	72.7 (8.8)	3.7 (1.0)	1.2 (0.5)	1.0 (0.4)	2.1 (0.7)	3.5 (0.5)	7.8 (4.2)
Never recovered	37	117.3 (10.3)	71,3 (8.3)	3.7 (1.0)	1.1 (0.5)	1.1 (0.4)	2.1 (0.8)	3.6 (0.6)	8.0 (3.3)

^a^Experienced both wasting and stunting.

### Factors associated with cardiovascular risk factors and schooling levels

Tables 3 and 4 show a summary of generalised linear regression analysis results to predict independent influence of childhood undernutrition on adolescent blood pressure and schooling achieved after adjusting for recorded socio-demographic, dietary, and lifestyle factors.

**Table 3.** Association between childhood stunting and blood pressure, and school achievement during adolescence (multivariate analysis).

		Systolic blood pressure (mmHg)		Diastolic blood pressure (mmHg)		Education attained (years)	
Characteristic		Coefficient (95%CI)	p-value	Coefficient (95%CI)	p-value	Coefficient (95%CI)	p-value
**Height- for- age change**							
Normal		Ref		Ref		Ref	
Stunted all through		-2.90 (-6.41, 0.61)	0.10	-0.23 (-2.70, 2.22)	0.85	-2.82 (-5.48, -0.70)	0.04
Stunted but recovered		0.67 (-1.66, 3.00)	0.57	0.19 (-1.55, 1.92)	0.83	-1.04 (-2.97, 0.90)	0.29
Normal but later stunted		0.99 (-1.84, 3.84)	0.49	0.15 (-1.56, 1.87)	0.86	-0.12 (-2.09, 0.86)	0.90
**Age groups (years)**							
13-14		Ref		Ref		Ref	
15-16		0.67 (-1.66, 3,00)	0.57	-0.43 (-2.10, 1.23)	0.61	3.75 (1.91,5.56)	<0.01
17-19		0.99 (-0.92, -1.84)	0.49	0.60 (-1.35, 2.50)	0.54	4.91(2.79,6.87)	<0.01
**Sex**							
Male		Ref		Ref			
Female		-0.36 (-2.23, 1.50)	0.70	2.20 (0.69, 3.72)	0.01	0.87 (-0.69, 2.42)	0.28
**Education**							
Incomplete primary		Ref		Ref			
Completed primary		2.13 (-0.59, 4.85)	0.12	0.02 (-1.89, 1.94)	0.98		
Post primary		-0.06 (-2.51, 2.46)	0.98	-0.42 (-2.2, 1.37)	0.65		
**Tribe**							
Muganda		Ref		Ref		Ref	
Other		-2.14 (-4.40, 0.12)	0.06	-0.24 (-1.94, 1.46)	0.27	1.17 (-0.61, 2.95)	0.19
**Wall type**							
Concrete and brick		Ref		Ref		Ref	
Mud and brick		1.49 (-1.38, 4.37)	0.31	-1.18 (-2.91, 0.54)	0.17	0.08 (-1.98,-2.16)	0.87
Mud and pole		0.65 (-1.44, 2.85)	0.54	-0.28(-1.83, 1.27)	0.72	0.23 (-1.43, 1.90)	0.78
**Reported alcohol consumption**							0.29
No	Ref			Ref			
Yes	6.57 (2.30, 10.84)		<0.01	2.34 (-0.37, 5.05)	0.09	Ref -1.15 (-3.30, 1.01)	0.29
**Physical activity per week**							
None	Ref			Ref		Ref	
1-3 times	1.17 (-1.51, 3.86)		0.39	0.33 (-1.50, 2.17)	0.72	-1.38 (-3.38, 0.62)	0.17
4 or more times	0.12 (-2.58, 2.83)		0.92	1.34 (-0.57, 3.26)	0.17	-2.28 (-4.42, -0.14)	0.04

**Table 4.** Association between childhood wasting and blood pressure, and school achievement during adolescence (multivariate analysis).

	Systolic blood pressure (mmHg)		Diastolic blood pressure (mmHg)		Education attained (years)	
Characteristic	Coefficient (95%CI)	p-value	Coefficient (95%CI)	p-value	Coefficient (95%CI)	p-value
**Weight- for- height change**						
Normal	Ref		Ref		Ref	
Wasted all through	-7.90 (-14.52, -1.28)	0.02	-3.92 (-7.42, -0.38)	0.03	-1.91 (-4.51, 0.68)	0.04
Wasted but recovered	-0.12 (-2.52, -2.27) 0.02	0.02	1.93 (0.11, 3.74)	0.04	-2.05 (-3.30, -0.79)	<0.01
Normal but later wasted	-1.88 (-3.97, 0. 21)	0.08	-0.77 (-2.11, 0.26)	0.28	-1.50 (-3.17, 0.18)	0.08
**Age groups (years)**						
13-14	Ref		Ref			
15-16	1.42 (-0.85, 3,70)	0.22	-0.16 (-1.70, 1.38)	0.84	3.71 (2.14,5.28)	<0.01
17-19	190 (-0.92, 4.71)	0.19	0.61 (-1.28, 2.50)	0.53	4.99 (3.11,6.87)	<0.01
**Sex**						
Male	Ref		Ref			
Female	-0.60 (-2.39, 1.18)	0.51	1.78 (0.36, 3.21)	0.01	0.91 (-0.37, 0.16)	0.16
**Education**						
Incomplete primary	Ref		Ref		Ref	
Completed primary	2.22 (-0.52, 4.96)	0.11	0.19 (-1.67, 2.05)	0.84		
Post primary	0.06 (-2.37, 2.49)	0.96	-0.26 (-2.02, 1.48)	0.76		
**Tribe**						
Muganda	Ref		Ref		Ref	
Other	-2.68 (-4.99, -0.45)	0.02	-0.85 (-2.36, 0.66)	0.27	1.13 (-0.81, 3.07)	0.25
**Wall type**						
Concrete and brick	Ref		Ref		Ref	
Mud and brick	2.30 (-0.52, 5.12)	0.11	-0.14 (-1.79, 1.50)	0.86	0.16 (-1.80,-2.11)	0.87
Mud and pole	0.74 (-1.34, 2.85)	0.49	-0.25 (-1.74, 1.24)	0.74	0.40 (-1.19, 2.00)	0.61
**Reported alcohol consumption**						
No	Ref		Ref		Ref	
Yes	4.51 (0.46, 8.57)	0.02	1.31 (-1.13, 3.99)	0.28	-1.64 (-3.45, 0.15)	0.07

### Association of height-for-age change (stunting) with blood pressure and schooling (multivariate analysis)

Compared to children who remained normal, children who remained stunted up to adolescence achieved less schooling years (−2.82, 95%CI [−5.48 -, −0.70] p = 0.04) and had some evidence of a borderline negative correlation between stunting and systolic blood pressure (−2.90, 95%CI [−6.41, 0.61] p = 0.10). Children who reported physical activity at least four times a week achieved less years of schooling (−2.28 [−4.42, −0.14] p = 0.04). Increase in both systolic and diastolic blood pressure was associated with alcohol consumption (6.57 95%CI [2.30, 10.84] p < 0.01 and 2.34 95%CI [−0.37, 5.05] p = 0.09) respectively. Girls had a higher mean diastolic pressure than boys (2.20, 95%CI [0.69, 3.72] p = 0.01) (Table 3).

### Association of weight-for-height (wasting) change with blood pressure and schooling (multivariate analysis)

Compared to children who were normal in the entire follow-up, those who were wasted from childhood to adolescence had lower mean systolic blood pressure (−7.90 95%CI [−14.52–1.28], p = 0.02), lower mean diastolic pressure (−3.92 95%CI [−7.42, −0.38], p = 0.03) and had fewer schooling years (−1.91 95%CI [−4.51, 0.68] p = 0.04). Those who recovered from wasting had higher mean blood pressure compared to the normal children (1.93, 95%CI (0.11, 3.74] p = 0.04) but lower school achievement (−2.05 95%CI [−3.30, −0.79] p < 0.01). Becoming wasted later in life was associated with lower systolic pressure and less schooling. In the final model we also found that belonging to a tribe other than the indigenous (Muganda) was associated with lower mean systolic blood pressure (−2.68 95%CI [−4.99, −0.45] p = 0.02) and alcohol consumption was associated with higher mean systolic blood pressure (4.51, 95%CI [0.46, 8.57] p = 0.02). Alcohol consumption had a borderline negative correlation with school achievement (−1.64, 95%CI [−3.45, 0.15] p = 0.07) (Table 4).

## Discussion

We found that in rural Uganda, more than two-thirds of the adolescents had experienced at least one form undernutrition; 10% remained stunted throughout follow-up and one third who were stunted before the age of 5 years had recovered from stunting and another one third who were normal before the age of five years became stunted by adolescence. Although wasting was less common, 1.3% remained wasted throughout the follow-up period, 15.6% who were wasted before 5 years of age had recovered from wasting and one third who were normal before the age of 5 years had become wasted later in adolescence.

Children who were wasted or stunted throughout the follow-up period had lower blood pressure levels compared to normal children, so undernutrition did not stand out as be a risk factor for later cardiovascular disease in contrast to earlier findings from elsewhere [2,22,23]. However, we observed that recovery from wasting was positively associated with an increase in diastolic pressure. This observation fits in with the idea that recovery from undernutrition is more important for cardiovascular risk development than is undernutrition itself as observed in a Finish birth cohort followed from 1944 to 2005 in which coronary heart disease was predicted by the tempo of childhood weight gain rather than by body size at any particular age [23]. Schulz, in 2010 also observed that the abrupt and large change in nutritional conditions after exposure to a prolonged period of nutritional stress plays a more important role in cardiovascular disease risk profile than the nutritional deprivation at any particular age [29]. It is interesting in this context that similar observations were made in the Leningrad Siege Study [30]. The Leningrad Siege Study found no difference in glucose tolerance, blood pressure and lipid concentration in adulthood between the subjects exposed and unexposed to starvation in utero or during infancy. In contrast, the Dutch Hunger study with a similar design found a strong link between early exposure to starvation and adult cardiovascular risk [31]. One major difference between these two populations was that the Russians in the Leningrad Siege Study continued to experience shortage of food while the Dutch in the Dutch Hunger study were able to quickly return to a normal dietary intake. Our observations support a similar discrepancy between groups sharing one and the same environment. Similar to our findings, Lule and colleagues found that postnatal weight gain rather birthweight was associated with higher blood pressure among adolescents in an urban setting in Uganda [32].

We also found that undernutrition in childhood correlated with lower school achievement during adolescence, even after controlling for age and socio-economic status. This is consistent with a number of studies that have shown that undernutrition has a negative influence on school grade attainment, cognitive development and social status in general [33–40]. Some mechanisms such as pathological changes to the brain resulting in structural permanent damage have been cited as possible mechanisms for lower school achievement despite recovering from undernutrition [18].

These findings together emphasize the magnitude of the long-term effects of early childhood undernutrition on later life particularly in rural Africa where undernutrition is common. In the entire Africa region, Eastern Africa has the highest levels of stunting with approximately 40% of children less than five years stunted. The number of stunted children in this region is projected to rise to 25 million by 2025 [41]. In rural Uganda, 50% of adolescents experienced undernutrition in their pre-school years [42].

It is particularly worrying that several children in rural communities in LMICs are exposed to undernutrition in their early years and end up with lower educational attainment which may lead to lower economic productivity, perpetuating a vicious cycle of poverty and undernutrition [8]. To break this cycle, there is need for concerted efforts at both national and local community levels to address undernutrition. The observation that recovery from wasting is associated with a rise in high blood pressure is important for LMICs because adolescents who were born when undernutrition rates were high are now exposed to a rapidly changing food environment characterized by energy-dense foods, have a risk of developing chronic diseases. Thus, prevention and prompt management efforts for child undernutrition in LMICs is a long-term investment that governments need to seriously consider to save the future generation from chronic diseases and poverty. School feeding programs in Uganda have been shown to improve performance in schools [43,44] mainly through improved attendance. There is need to strengthen the school feeding policies to include feeding in early years of learning to address long-term effects of early childhood undernutrition.

We found other factors to be independently associated with higher blood pressure among adolescents. For example, female gender and alcohol consumption were associated with higher mean blood pressure. Adolescent girls have a higher mean blood pressure and better school achievement perhaps because hormonal changes in adolescence occur earlier in girls resulting in an earlier growth spurt than in boys. For the same reason, girls have faster physical growth and cognitive development than boys.

As observed earlier in the same population among adults, individuals from tribes that migrated into the study area had lower levels of blood pressure compared to the indigenous tribe [45]. These ethnic differences may be explained by genetic differences yet to be explored in this context.

One strength of our study is its longitudinal design that gives a more robust link between early childhood exposures and adolescent health in rural settings where undernutrition is common yet its effects are largely understudied. We had intermediate measurements of weight and height that enabled us to determine the change in nutritional status as the determinant of cardiovascular disease risk and educational achievement other than a single measurement at a time. The availability of socio-economic, dietary and lifestyle data collected in our study also enabled us to account for some confounding.

Our study had some limitations. We only examined long-term effects of childhood undernutrition among children who survived and were present in the study villages after a decade of follow-up. Those who migrated could potentially be with higher socio-economic status with higher risk of cardiovascular diseases leading to an underestimate of the true effects. However, we still had close to 80% of the children available thus making our findings generalizable in the majority of the children in this population. Additionally, we did not have sufficient variables to measure socio-economic status since most of the asset indices measured were homogeneously distributed across the entire population; hence, the choice of wall material which seemed to stratify the population wealth status fairly well.

We could not ascertain if undernutrition resulted from a low birth weight or later since we did not have access to birthweight data. In this population, most children are not weighed at birth because they are delivered at home and for those who are delivered at health facilities, linkage of birth records with the survey data is not possible since there are no common identifiers between health facility data and our population level data. However, a recent publication in another urban cohort in Uganda showed no association between low birthweight and blood pressure but rather an increase in blood pressure with post-natal weight gain as in our study [32].

In conclusion, this study suggests the effect of childhood undernutrition on blood pressure is largely dependent on recovery from wasting rather than just an episodic experience in early childhood while educational achievement is compromised regardless of whether recovery from undernutrition happens. There is thus a continued need to assess anthropometry in childhood especially among pre-school children and a need to invest in the follow-up and screening of children who recover from wasting for the purpose of early detection and management of risk factors for cardiovascular disease. Our results re-echo the long-term effects of undernutrition on health and educational achievement that should make improvement of early childhood nutrition in rural parts of Uganda where most children live, a high policy priority. A possible entry point is through improvement of school policies targeting feeding of pre-school children in day-care learning centres. We recommend regular follow-up and additional assessment of this cohort of adolescents for chronic diseases in later adult life.

## Paper context

Little is known about the relationship between early undernutrition and health effects on later life in low- and middle-income countries where the burden of undernutrition is high. We investigated this in a rural Ugandan population and found that recovery from wasting is associated with a rise in blood pressure while both wasting and stunting regardless of recovery were associated with low educational achievement. Interventions preventing undernutrition are needed to address additional long-term effects.
